# New Drugs, Appliances, and Things Medical

**Published:** 1891-10-31

**Authors:** 


### NEW DRUGS, APPLIANCES, AND THINGS MEDICAL.

[All preparations, appliances, novelties, etc., of which a notice is desired, should be sent for The Editor, to care of The Manager, 140, Strand, London, W.C.]

### FRAME FOOD.

The analysis of frame food proves it to be wholesome and nutritious, and of excellent composition for easy digestion, and the building up of the tissues. We have, for ourselves, made a somewhat extended trial of it, in the nursery and at the breakfast table. It is our purpose also to try it more extensively in the sick room. The food is admirable for the nursery, because it is so exceedingly palatable. The writer's little boy, a sturdy Briton of two years old, showed such a very determined liking for it a month or six weeks ago, that the writer out of curiosity had a plate prepared for himself. He considered the taste delicious, and was, indeed, as fond of it as the boy. There is no doubt that if the frame food continues to be produced in flavour and character equal to sample it has a widespread reputation and sale before it.

